# Deletion of Glutamate Delta-1 Receptor in Mouse Leads to Aberrant Emotional and Social Behaviors

**DOI:** 10.1371/journal.pone.0032969

**Published:** 2012-03-07

**Authors:** Roopali Yadav, Subhash C. Gupta, Brandon G. Hillman, Jay M. Bhatt, Dustin J. Stairs, Shashank M. Dravid

**Affiliations:** 1 Department of Pharmacology, Creighton University, Omaha, Nebraska, United States of America; 2 Department of Psychology, Creighton University, Omaha, Nebraska, United States of America; University of Victoria, Canada

## Abstract

The delta family of ionotropic glutamate receptors consists of glutamate δ1 (GluD1) and glutamate δ2 (GluD2) receptors. While the role of GluD2 in the regulation of cerebellar physiology is well understood, the function of GluD1 in the central nervous system remains elusive. We demonstrate for the first time that deletion of GluD1 leads to abnormal emotional and social behaviors. We found that GluD1 knockout mice (GluD1 KO) were hyperactive, manifested lower anxiety-like behavior, depression-like behavior in a forced swim test and robust aggression in the resident-intruder test. Chronic lithium rescued the depression-like behavior in GluD1 KO. GluD1 KO mice also manifested deficits in social interaction. In the sociability test, GluD1 KO mice spent more time interacting with an inanimate object compared to a conspecific mouse. D-Cycloserine (DCS) administration was able to rescue social interaction deficits observed in GluD1 KO mice. At a molecular level synaptoneurosome preparations revealed lower GluA1 and GluA2 subunit expression in the prefrontal cortex and higher GluA1, GluK2 and PSD95 expression in the amygdala of GluD1 KO. Moreover, DCS normalized the lower GluA1 expression in prefrontal cortex of GluD1 KO. We propose that deletion of GluD1 leads to aberrant circuitry in prefrontal cortex and amygdala owing to its potential role in presynaptic differentiation and synapse formation. Furthermore, these findings are in agreement with the human genetic studies suggesting a strong association of GRID1 gene with several neuropsychiatric disorders including schizophrenia, bipolar disorder, autism spectrum disorders and major depressive disorder.

## Introduction

The delta family of ionotropic glutamate receptors (iGluRs) consists of the glutamate δ1 (GluD1) and glutamate δ2 (GluD2) receptors which share ∼60% homology [1]. GluD2 is selectively expressed at the parallel fiber-Purkinje cell (PF-PC) synapse in the adult cerebellum [2]–[4]. Deletion of GluD2 leads to severely reduced PF-PC synapses and abrogated long-term depression together with ataxia and impaired motor learning [5]. Moreover, recent studies have established that the N-terminal domain (NTD) of GluD2 is necessary and sufficient for induction of presynaptic differentiation and synapse formation [6]–[8]. GluD1 is highly expressed in the inner hair cells of the organ of Corti [9], [10]. Deletion of GluD1 leads to a deficit in high frequency hearing in mice [10]. In the central nervous system (CNS), GluD1 is expressed diffusely throughout the forebrain during early development [1], [11] however its functional significance remains elusive. GluD1 knockout mice (GluD1 KO) have normal learning in the water maze test and intact hippocampal long-term potentiation [10]. *In vitro* studies indicate that GluD1, similar to GluD2, may induce presynaptic differentiation and synapse formation [6], [8], [12], [13].

The strongest evidence of potential involvement of GluD1 in regulating neural circuitry comes from human genome-wide association studies. Genetic association studies have established the GRID1 gene, which codes for GluD1, as a strong candidate gene for schizophrenia, bipolar disorder, and major depressive disorder [14]–[21]. Copy number variation studies have also implicated GRID1 in autism spectrum disorder (ASD) [22]–[24]. In addition, GRID1 gene is localized to the 10q22–q23 genomic region which is a site for recurrent deletions associated with cognitive and behavioral abnormalities [25], [26].

In this study we performed behavioral characterization of the GluD1 KO mice and found remarkable features including hyperactivity, lower anxiety-like behavior, depression-like behavior, hyperaggression and deficits in social interaction in the GluD1 KO. Additionally we found changes in synaptoneurosomal expression of synaptic proteins, including iGluR subunits, in the prefrontal cortex and amygdala of GluD1 KO. The synaptoneurosomal abnormalities further support a potential role of GluD1 in the regulation of synapse via its proposed effect on presynaptic differentiation and synapse formation [6], [8], [12], [13]. Overall, our results underscore the importance of GluD1 in development of normal neural circuitry in brain regions that regulate emotional and social behaviors, dysregulation of which may lead to appearance of characteristic features of neuropsychiatric disorders.

## Materials and Methods

### Ethics statement

All experimental protocols were approved by the Creighton University Institutional Animal Care and Use Committee Policies and Procedures. In this study strict measures were taken to minimize pain and suffering to animals in accordance with the recommendations in the Guide for Care and Use of Laboratory Animals of the National Institutes of Health. The IACUC protocols for these studies were 0893 and 0865.

### Generation and genotyping of GluD1 knockout mice

GluD1 KO mice were obtained from Dr. Jian Zuo, St. Jude's Children's Hospital [10]. These mice had been generated by creating a targeting construct that deleted exons 11 and 12 of the GluD1 gene (GRID1). The targeted disruption ensured removal of three of the four transmembrane domains of the GluD1 receptor and introduced a frameshift after exon 12. In the PCR analysis no 220 bp wildtype bands (in the deleted region) were detected in the homozygous GluD1 KO mice. All mice analyzed were from a mixed background of 129/SvEv and C57BL/6 in the F2 to F6 generations [10].

Genotyping was done as previously described [10]. The primers used for the reaction were as follows: a pair of primers from the deleted region of GluD1; 5′GCAAGCGCTACATGGACTAC 3′ and 5′GGCACTGTGCAGGGTGGCAG 3′ and a pair of primers from the targeting vector; 5′CCTGAATGAACTGCAGGACG 3′ and 5′CGCTATGTCCTGATAGCGATC 3′.

### Mouse husbandry

Wildtype (WT), GluD1 heterozygous and GluD1 KO male mice, aged 8 weeks were either group housed (4–5 mice) or single housed (as per the requirements of the test) in the animal house facility at a constant temperature (22±1°C) and a 12-hr light-dark cycle with free access to food and water. Behavioral testing was performed between 9:00 a.m and 4:00 p.m. The study did not involve using female mice to avoid the confounding effects of the estrus cycle on behavioral and neurochemical measures. The WT, GluD1 heterozygous and GluD1 KO mice were obtained from previously genotyped parent cages.

### Behavioral testing

Behavioral testing was performed between 9:00 a.m and 4:00 p.m. As per the requirements of the tests, mice were handled for 3 days to acclimatize them to the experimenter before subjecting them to the experimental procedures. Each day the experimenter picked up each animal and the animal was allowed to explore the experimenter's hand for 2 min. All experimental animals were placed in the experimental room at least 60 min before beginning any experimental protocol. Unless indicated otherwise, all experimental environments were thoroughly cleaned with 70% ethanol between trials which was given time to dry away. All behavioral procedures were video-recorded and scored by a scorer blind to the genotype of the animal via a random coding system of the video files.

### Lithium treatment

Chronic lithium treatment was conducted as previously described [27]. This protocol has been shown to maintain trunk blood level of lithium within the human therapeutic level (0.4–1.2 mM). We measured the blood lithium concentration at the end of the experiments and found that they were within this stipulated range (0.7–1.0 mM) (performed at Creighton University Medical laboratory, Omaha, NE, USA). Lithium concentration was measured using the Thermo Scientific Infinity Lithium reagent, Rockford, IL, USA. The assay is based on change in absorbance with binding of lithium to substituted porphyrin compound at alkaline pH. Absorbance was measured using Beckman Coulter DXC Synchron, Brea, CA, USA. WT and GluD1 KO mice were fed lithium carbonate chow (2.4 g/kg, Bioserve, Frenchtown, NJ, USA) or control chow identical to lithium carbonate chow with the exception of lithium salt for 4 weeks. Due to the side effects of polyuria and polydipsia the cage bedding and water bottles were changed twice a week. Mice were also supplied with 0.9% NaCl in addition to tap water to supplement for possible electrolyte imbalance. We measured the body weight and food consumption in a small group of animals during the lithium treatment. The body weight did not decrease over the course of lithium diet and the food consumption was also similar to the regular diet. Mice were then subjected to the open field, forced swim and the resident-intruder test, the procedures for which have been described later in the methods section.

### D-Cycloserine (DCS) treatment

There were four groups of mice, WT saline, GluD1 KO saline, WT DCS and GluD1 KO DCS. DCS (Sigma-Aldrich (C6880); St Louis, MO, USA) was dissolved in 0.9% saline. Freshly dissolved DCS was used for experiments. We used a dose of DCS that has previously been shown to be efficacious in social behavior in mice [28], [29]. Mice were administered a single dose of 320 mg/kg DCS in 0.9% saline (80–100 µl) or 0.9% saline (90 µl) intraperitoneally, 20 min prior to beginning the sociability/social novelty test. The sociability test and the test for social novelty were performed after DCS treatment. The procedures for the sociability test and the test for social novelty are described later in the methods section.

### Test for vision

To test normal vision in GluD1 KO we performed a qualitative test. The mice were picked up by the tail and slowly lowered to a wire cage lid. Vision (score of 1) was reflected in an animal's extending its forepaws and attempting to grip the lid just before contact with the surface. If the mouse failed to exhibit this behavior it was scored as blind (score of 0). Since all animals in both genotypes WT and GluD1 KO received a score of 1 no statistical analysis was performed.

### Test for olfaction

Hide the cookie test was performed to test olfaction [30]. Two days before the test, mice were acclimatized to the food stimulus; a mini cookie from Teddy Grahams (Nabisco, Hanover, New Jersey). 18–24 hrs before the test, mice were fasted with free access to water. The mouse to be tested was then placed in a clean cage with 3 cm deep bedding and allowed to acclimate to the cage for 5 min. The mouse was then transferred to an empty cage. In the cage containing the bedding, the food stimulus was buried 1 cm beneath the bedding in a random corner of the cage. The bedding surface was made smooth. The mouse was reintroduced into the cage and the latency to find the food stimulus was recorded. In case a mouse failed to find the buried food stimulus, the mouse was allowed to explore the cage for 15 min and the latency was scored as 900 sec.

### Open field test

The open field test was performed as previously described [31]. Spontaneous locomotor activity was recorded in an open field 25×25 cm arena with grid marking (6.25×6.25 cm) on the bottom. White light of 300–330 lux intensity was used to evenly illuminate the entire open field arena. The number of times and total time the mouse entered the central four squares (central field penetration) as well as the total number of line crosses made by the mouse in the 15 min interval were tallied and expressed as cumulative line crosses during the 15 min interval. Scoring was done by an experimenter blind to the genotype of the mice.

### Marble burying test

The marble burying test was performed as previously described [32] with minor modifications. Anxiety-like behavior was assessed with the marble burying test using a square 25×25 cm arena with the home cage bedding to a height of 5 cm. White light of 300–330 lux intensity was used to evenly illuminate the entire arena of the marble burying test. 36 dark colored marbles 1.5 cm in diameter were placed 5 cm apart. The test mouse was placed in the same corner of the open field arena and left in the arena for 30 min. At the end of the 30 min the mouse was taken out and the number of marbles buried was counted. If two-thirds of a marble was buried inside the bedding it was counted as buried otherwise non-buried. The marble burying test is based on the observation that mice experiencing greater anxiety-like behavior would bury greater number of marbles [32].

### Elevated plus maze

The elevated plus maze was constructed and conducted as previously described [33] with modifications for time similar to [31]. The apparatus was made of plastic material and was elevated to a height of 40 cm above the ground. Each arm of the plus maze was 30 cm in length and 5 cm in width. Additionally, the closed arms had wall enclosures that were 15.25 cm high. The central platform was a square of 5×5 cm. Light intensity around the maze was set at 300–330 lux. Mice were placed on the elevated plus maze for 15 min. Mice were placed on the elevated plus maze facing the open arm opposite to the experimenter. Arm location was recorded with an elevated video camera. The animals were videotaped for 15 minutes. The number of entries and the time spent in the open and closed arms were recorded over the entire duration of the test.

### Forced swim test

The forced swim test measures depression-like behavior in mice [34] with modifications. Mice were placed in a glass cylinder 13 cm diameter X 24 cm high, filled with 10 cm high water (22±2°C), for a period of 5 min. Water was changed between subjects. All test sessions were recorded by a video camera positioned in front of the glass cylinder. Videotapes were subsequently scored by an observer blind to the genotype of the mice. Mice were judged immobile when they remained floating passively in the water, with minor movements to keep their heads above the water. The videos were scored for total duration of immobility and latency to immobility. Latency to immobility is defined as the duration of time from the beginning of the test session, to the appearance of the first 3 sec immobile event.

### Sucrose preference test

The sucrose preference test was conducted as previously described [35] with minor modifications. Mice were individually housed for this test. During the test, mice were given for 24 hr, a free choice between 1% sucrose in one bottle and tap water in another. To avoid the possible effects of side preference in drinking behavior, the position of the bottles was interchanged after 12 hr. The mice were not subjected to previous food or water deprivation prior to the test. Water and sucrose consumption was calculated in both groups by weighing the bottles. The preference for sucrose was calculated as a percentage of sucrose solution consumed of the total amount of liquid drunk.

### Resident-intruder test

The resident-intruder test was performed as previously described [36] with minor modifications. The resident animals were singly housed for three weeks and the last one week prior to conducting the test no cage change was done. A WT mouse (intruder) was introduced into the home cage of the singly housed resident animal. The grid wire holding the feed and the water bottle was removed; the cotton in the cage was also removed while conducting the test. All the resident animals weighed 4–5 g more than the intruder animals. The two mice were left to interact for 10 min. Each intruder mouse was only used once. The interaction between the two mice was videotaped and scored later for attack latency and frequency of attacks with the scorer being blind to the genotype of the mice. Attack latency is defined as the time taken for the resident mouse to initiate the first attack.

### Sociability test/Preference for social novelty

The procedure for the social interaction and social novelty test were performed as previously described [37], [38] with minor modifications. The experiment was conducted in a room with a light intensity of 300–330 lux. The social interaction chamber is a three chambered apparatus made of clear Plexiglas. Doorways built into the two dividing walls controlled access to the side chambers. Each of the three chambers was 20 cm length ×40.5 cm width ×22 cm high. In the sociability test, the experimental mouse was subjected to a 5 min acclimation period in the middle chamber with doors to both side chambers closed. This was done in the presence of an unfamiliar adult male (Stranger 1) in one of the chambers and an inanimate object in the other chamber. A transparent plastic container with holes was used to enclose the Stranger 1 mouse. The other chamber contained an empty container of the same size (referred to as inanimate object). A weighted plastic cup was placed on top of each plastic container to prevent the subject from climbing to the top. Location of the stranger mouse and the inanimate object was alternated between the two-side chambers on consecutive sessions. During the sociability test doors to the chambers on either side were opened and the experimental mouse was allowed to explore the three chambers for 10 min. A circle with a 1 cm radius was marked around the periphery of the plastic container. The duration the experimental mouse spent within this circle interacting with the inanimate or Stranger 1 containing plastic container was recorded. The percent time interacting with Stranger 1 was reported as; time interacting with Stranger 1/[(time interacting with the inanimate object) + (time interacting with Stranger 1)]*100. Subsequently the same experimental mouse was subjected to the test for social novelty, beginning with the experimental mouse being acclimatized to the middle chamber for 10 min in the presence of the two stranger mice, one on either side, with the second unfamiliar mouse being a new stranger mouse (Stranger 2) placed in the opposite side, which was previously empty during the sociability test with the doors on either side of the middle chamber to the side chambers being closed. During the test for social novelty, the doors on either side were raised open and the experimental mouse was allowed to explore all the three chambers for 10 min. The duration the experimental mouse spent exploring the circle around plastic container containing Stranger 1 or Stranger 2 was recorded. The percent time interacting with Stranger 2 was reported as; time interacting with Stranger 2/[(time interacting with Stranger 1 + time interacting with Stranger 2)]*100. All the stranger mice used for the experiment were WT mice. The entire procedures were video-taped and scored later by a person blind to the genotype of the mice.

### mRNA expression analysis

For mRNA expression analysis 15 day old naive WT and GluD1 KO mice were anesthetized using isoflurane anesthesia. Mice were then decapitated and thereafter all experimental procedures were conducted on ice. The brain was dissected out, the amygdala, prefrontal cortex and hippocampi were crudely dissected out and the freshly dissected amygdala, prefrontal cortex and hippocampi were put into TRIzol reagent (1 ml TRIzol reagent per 50–100 mg of tissue) and homogenized. Phase separation was achieved by addition of 0.2 ml of chloroform and centrifugation at 12,000× g for 15 min at 2–8°C. For RNA precipitation, the aqueous phase was transferred into a fresh tube and 0.5 ml isopropyl alcohol was added. The samples were incubated for 10 min and centrifuged at 12,000× g for 10 min at 2–8°C. RNA pellet was washed once with 75% ethanol, briefly dried and dissolved in DEPC water.

The primers used for the reaction were as follows: GluD1 forward 5′ ACCTCCTGGAATGGGATGAT, GluD1 reverse 5′ CCTCAGGCTTCTTGATGAGG, β-actin forward 5′AATTTCTGAATGGCCCAGGT, β-actin reverse 5′ TGTGCACTTTTATTGGTCTCAA. For RT-PCR, 3 µg RNA was purified from the amygdala, hippocampus and the prefrontal cortex of postnatal day 15 WT and GluD1 KO mice and subjected to DNAse treatment and was thereafter reverse transcribed into cDNA in a 10 µl reaction and the cDNA thus obtained was subjected to a conventional RT-PCR . In each case, RNA samples were verified as free of DNA contamination by running RT-PCR negative control lacking reverse transcriptase. From the 10 µl cDNA, 5 µl was used for PCR reaction with GluD1 primers and 5 µl was used for PCR reaction with β-actin primers. For the PCR, total of 32 cycles were employed with annealing at 55°C and extension at 72°C. 12 µl of each sample's PCR product was separated by 2% agarose gel electrophoresis and the gels were imaged using a UV image analyzer.

### Synaptoneurosome preparation and western blot analysis

For synaptoneurosomal preparation 45–50 day old naive WT and GluD1 KO mice were anesthetized using isoflurane anesthesia, mice were then decapitated and thereafter all experimental procedures were conducted on ice. The amygdala and prefrontal cortex were crudely dissected out and put into synaptoneurosomal buffer at 4°C. Thereafter, the fresh tissue, amygdala and prefrontal cortex were used for synaptoneurosome preparation and western blotting.

The freshly isolated amygdala and prefrontal cortex from WT and GluD1 KO mice were homogenized in synaptoneurosome buffer (10 mM HEPES, 1 mM EDTA, 2 mM EGTA, 0.5 mM DTT, 10 µg/ml leupeptin, and 50 µg/ml soybean trypsin inhibitor, pH 7.0) as previously described [39], additionally containing 5 mg/ml pepstatin, 50 mg/ml Aprotonin and 0.5 mM phenylmethanesulfonylfluoride (PMSF). From this step forward the homogenate was kept ice-cold at all times to minimize proteolysis throughout the isolation procedure. The homogenate was diluted further with the same volume of synaptoneurosome buffer and briefly and gently sonicated delivering 3 pulses using an output power of 1 Sonic dismembrator Model 100 (Fischer Scientific, NJ, USA). The sample was loaded into a 1.0 ml Luer-lock syringe (BD syringes) and filtered twice through three layers of a pre-wetted 100 µm pore nylon filter CMN-0105-D (Small Parts Inc., Logansport, IN, USA) held in a 13 mm diameter filter holder XX3001200 (Milipore, MA). The resulting filtrate was loaded into a 1 ml Luer-lock syringe and filtered through a pre-wetted 5 µm pore hydrophilic filter CMN-0005-D (Small Parts Inc., Logansport, IN, USA) held in a 13 mm diameter filter holder. The resulting filtrate was centrifuged at 1000× g for 10 min. The pellet obtained corresponded to the synaptoneurosome fraction. Isolated synaptoneurosomes were resuspended in 75 µl of buffer solution containing 0.32 M sucrose, and 1 mM NaHCO_3_ (pH 7.0).

For western blotting synaptoneurosomes prepared from 45–50 day old naive WT and GluD1 KO mice were loaded on 10% Sodium dodecyl sulfate gel in equal amount (15 µg/well). The samples were run at 114 volts for a duration of 1 hr. Gels were transferred to nitrocellulose membrane (GE Healthcare, Piscataway, NJ, USA), a wet transfer was carried out. The voltage for transfer was kept at 114 volts and duration for which transfer was carried out was 1 hr 15 min. Electrophoresis and transfer apparatuses used were the Biorad mini protean tetra cell, from Bio-Rad Laboratories, Inc., Hercules, California, USA. Transfer was followed by blocking with 5% milk in Tris-buffered Saline with 1% Tween 20 (TBST) for 1 hr at room temperature. The primary antibodies GluA1 (Millipore, Billerica, MA, USA), 1∶1500; GluA2 (Millipore), 1∶2000; GluN2B (Millipore), 1∶1000; GluK2 (Abcam, Cambridge, MA, USA), 1∶1000; vesicular glutamate transporter 2 (vGluT2) (Millipore), 1∶1000; glutamic acid decarboxylase 67 (GAD67) (Millipore), 1∶1000; postsynaptic protein density 95 (PSD95) (Affinity Bioreagents, CO, USA), 1∶2500 and Synaptophysin (Zymed, Carlsbad, CA, USA), 1∶2500 were used and kept overnight for incubation at 4°C followed by washing and were incubated with horse-radish peroxidase conjugated anti-rabbit secondary antibody 1∶5000; (Cell Signaling Technology, Danvers, MA, USA) for 1 hr at room temperature followed by washing with TBST. Blots were developed using enhanced chemiluminescent (ECL) Plus Western Blotting Detection System kit RPN2132 (GE Healthcare, Piscataway, NJ, USA) and images were taken using Precision Illuminator Model B95 (Imaging Research Inc., Germany) with a MTI CCD 72S camera and analyzed using MCID Basic software version 7.0 (Imaging Research, St. Catharines, ON, Canada). The X-ray film processor used was model- BMI No 122106 (Brown's Medical imaging, Omaha, NE, USA). For analysis of protein expression, first, the optical density of each sample was normalized to β-actin. Thereafter, the optical density was normalized to the mean of the WT samples. The average ± SEM of optical densities of GluD1 KO samples, that were normalized to WT mean, are represented as Ratio (KO/WT) ± SEM. The P values were calculated from optical densities of WT and GluD1 KO samples normalized to the WT mean.

### Statistics

Data were analyzed using Student's unpaired t-test with Welch's correction (open field test, marble burying test, elevated plus maze test, forced swim test, sucrose preference test, resident-intruder test, sociability test, social novelty test, molecular changes in the amygdala and prefrontal cortex) or two-way ANOVA (analysis of variance) with Bonferroni's post-hoc test (lithium effect on open field test, forced swim test and resident-intruder test and DCS effect on sociability and social novelty test and protein expression in amygdala and prefrontal cortex). Differences were considered significant if P≤0.05. Prism 4 (GraphPad Software Inc., San Diego, CA, USA) was used for analysis and representation.

## Results

GluD1 KO mice have previously been described to be normal in motor coordination, balance and spatial learning [10]. A general observation we made with the GluD1 KO was their uneasiness during regular human handling displayed by their hypersensitivity to contact with the experimenter. We performed a set of experiments to further test for locomotor activity, anxiety-like behavior, depression- and aggression-like behaviors and social interaction, in GluD1 KO mice. Moreover, we tested the effect of lithium and DCS on the behavioral deficits in GluD1 KO.

### GluD1 knockout mice exhibit greater spontaneous activity

**Figure 1:**
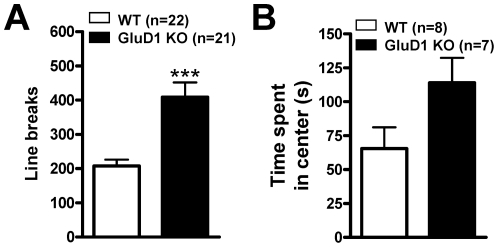
GluD1 KO have higher spontaneous activity. **A.** GluD1 KO mice (n = 21) displayed increased spontaneous locomotor activity in the 15 min open field test compared to the WT mice (n = 22) (unpaired t-test with Welch's correction, P = 0.0002, F = 5.226). **B.** GluD1 KO mice (n = 7) display a trend to spend greater time in the central field of the open field arena compared to WT mice (n = 8) (unpaired t-test, P = 0.0649, F = 1.206). Data are presented as mean ± SEM. *** represents P<0.001.

As a first step we assessed some of the sensory abilities in GluD1 KO since these may confound results obtained from other behavioral tasks. We measured the olfaction ability for WT (n = 8) and GluD1 KO (n = 7) and found no significant difference between the two genotypes (unpaired t-test, P = 0.7618, F = 1.185). GluD1 KO (n = 6 for both WT and GluD1 KO mice) had normal vision as seen from the results of the grip test for vision. We next examined the spontaneous activity in an open field test. Compared to the WT mice (n = 22), GluD1 KO mice (n = 21) displayed higher spontaneous locomotor activity (Fig. 1A; unpaired t-test with Welch's correction, P = 0.0002, F = 5.226). Both genotypes displayed habituation to the novel environment, as indicated by gradual reduction in activity in a time-dependent manner (data not shown). Additionally, we measured the time spent in the central square of the open field arena which is a measure of anxiety-like behavior. Mice displaying greater anxiety-like behavior tend to spend greater time in the periphery of the open field arena compared to the central square. GluD1 KO mice (n = 7) manifest a trend for spending greater time in the central field of the open field arena compared to WT mice (n = 8) (Fig. 1B; unpaired t-test, P = 0.0649, F = 1.206).

### GluD1 knockout mice exhibit lower anxiety-like behavior

**Figure 2:**
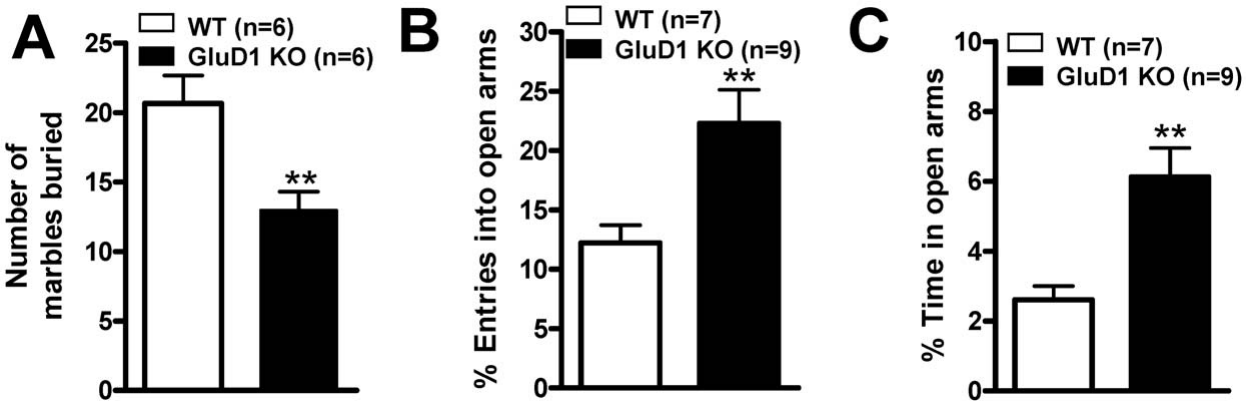
GluD1 KO manifest lower anxiety-like behavior. **A.** GluD1 KO mice (n = 6) buried significantly fewer marbles compared to WT mice (n = 6) (unpaired t-test, P = 0.0097, F = 2.333). **B.** GluD1 KO mice (n = 9) made significantly higher percent entries into the open arms compared to WT mice (n = 7) (unpaired t-test with Welch's correction, P = 0.0093, F = 4.621). **C.** GluD1 KO mice (n = 9) spent significantly greater percent time in the open arms compared to WT mice (n = 7) in the elevated plus maze test (unpaired t-test with Welch's correction, P = 0.0027, F = 5.746). Data are presented as mean ± SEM. ** represents P<0.01.

Based on the results of the time spent in the central square of the open field arena we found that GluD1 KO mice display a trend for lower anxiety-like behavior. In order to further study anxiety-like behavior, we conducted the marble burying test. GluD1 KO mice (n = 6) buried significantly fewer marbles compared to WT mice (n = 6) (Fig. 2A; unpaired t-test, P = 0.0097, F = 2.333). In addition, in the elevated plus maze GluD1 KO mice (n = 9) were found to have significantly higher percent entries into the open arms compared to WT mice (n = 7) (Fig. 2B; unpaired t-test with Welch's correction, P = 0.0093, F = 4.621) and GluD1 KO mice (n = 9) spent significantly greater percent time in the open arms compared to WT mice (n = 7) (Fig. 2B; unpaired t-test with Welch's correction, P = 0.0027, F = 5.746). Although the parameter of number of entries may be confounded by hyperactivity which was observed with GluD1 KO in the open field test, overall these results suggest that GluD1 KO mice manifest lower anxiety-like behavior.

### GluD1 knockout mice exhibit depression-like behavior

**Figure 3:**
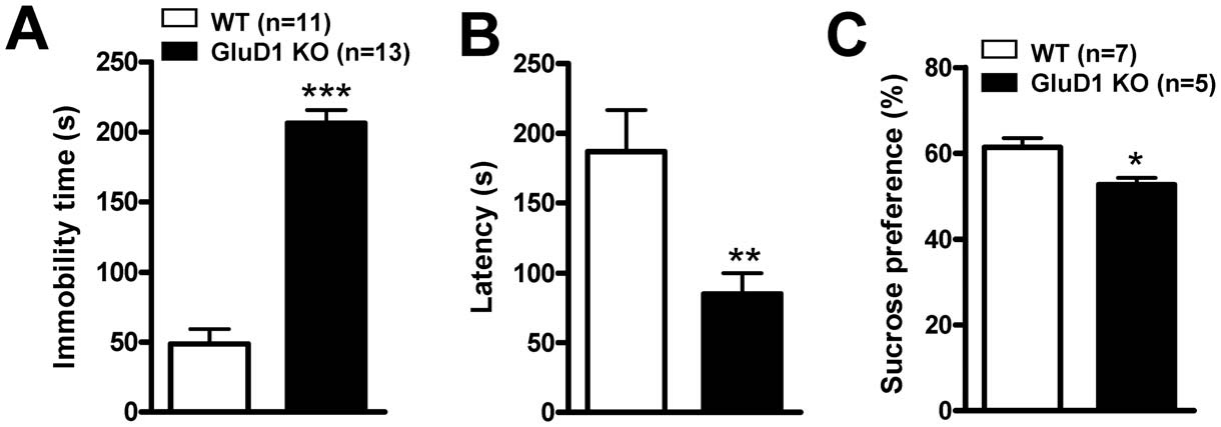
GluD1 KO exhibit depression-like behavior. **A.** In a forced swim test GluD1 KO (n = 13) displayed a greater duration of immobility compared to WT mice (n = 11) (unpaired t-test, P<0.0001, F = 1.060). **B.** GluD1 KO (n = 13) had a significantly shorter latency to immobility compared to WT mice (n = 11) (unpaired t-test, P = 0.0040, F = 3.446). **C.** GluD1 KO mice (n = 5) manifested significantly lower preference for sucrose compared to WT (n = 7) mice (unpaired t-test, P = 0.0118, F = 2.275). Data are presented as mean ± SEM. *** represents P<0.001 and ** represents P<0.01 and * represents P<0.05.

We next tested depression-like behavior in WT and GluD1 KO using the forced swim test. GluD1 KO mice (n = 13) displayed a significantly greater duration of immobility (Fig. 3A; unpaired t-test, P<0.0001, F = 1.060) compared to the WT (n = 11) mice. In addition, the latency was significantly shorter for GluD1 KO compared to WT (Fig. 3B; unpaired t-test, P = 0.0040, F = 3.446). Additionally we conducted the sucrose preference test to assess for depression-like behavior in GluD1 KO mice. GluD1 KO mice (n = 5) manifested significantly lower preference for sucrose compared to WT (n = 7) mice (Fig. 3C; unpaired t-test, P = 0.0118, F = 2.275).

### GluD1 knockout mice exhibit aggression-like behavior

**Figure 4:**
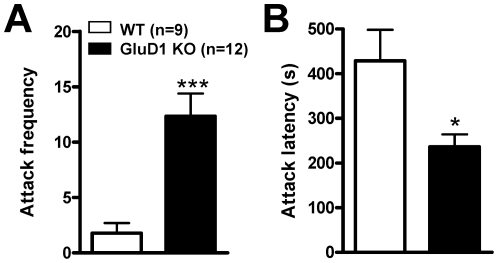
GluD1 KO are hyperaggressive. **A.** In the resident-intruder test for aggression GluD1 KO mice (n = 12) manifested robust aggression with a higher attack frequency compared to WT mice (n = 9) (attack frequency, unpaired t-test with Welch's correction, P = 0.0003, F = 6.577). **B.** GluD1 KO mice (n = 12) have a significantly shorter attack latency compared to WT mice (n = 9) (unpaired t-test, P = 0.0105, F = 4.605). Data are presented as mean ± SEM. *** represents P<0.001 and * represents P<0.05.

Another observation we made was that the GluD1 KO mice displayed more home cage fights when group housed (data not shown). Thus, in order to assess aggressive behavior in the GluD1 KO mice we conducted the resident-intruder test. The GluD1 KO mice (n = 12) attacked an intruder mouse with a significantly higher frequency than WT mice (n = 9) (Fig. 4A; unpaired t-test with Welch's correction, P = 0.0003, F = 6.577). GluD1 KO mice on an average attacked the intruder mouse twelve times as compared to the WT mice which attacked the intruder approximately twice in the 10 min duration of the resident-intruder test. In addition, GluD1 KO mice had a shorter latency to attack compared to the WT mice (Fig. 4B; unpaired t-test, P = 0.0105, F = 4.605).

### Behavioral effects of chronic lithium treatment on GluD1 knockout mice

**Figure 5:**
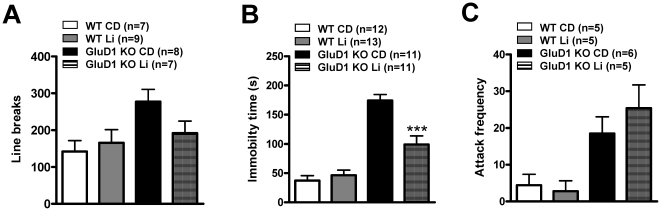
Lithium rescues depression-like behavior in GluD1 KO. **A.** Open field test was performed on four groups, control diet WT (n = 7), lithium WT (n = 9), control diet GluD1 KO (n = 8) and lithium GluD1 KO (n = 7). In the open field test no drug X genotype effect was observed in the number of line crossings (two-way ANOVA, drug F(1, 27) = 0.8275, P = 0.3710; genotype F(1, 27) = 5.678, P = 0.0245; interaction F(1, 27) = 2.589, P = 0.1192). **B.** Forced swim test was performed on control diet WT (n = 12), lithium WT (n = 13), control diet GluD1 KO (n = 11) and lithium GluD1 KO (n = 11). Lithium reduced the immobility time in forced swim test in the GluD1 KO (two-way ANOVA, drug F(1, 43) = 9.717, P = 0.0033; genotype F(1, 43) = 80.49, P<0.0001; interaction F(1, 43) = 15.84, P = 0.0003). **C.** Resident-intruder test was performed on control diet WT (n = 5), lithium WT (n = 5), control diet GluD1 KO (n = 6) and lithium GluD1 KO (n = 5). Lithium failed to rescue the higher attack frequency in GluD1 KO in the resident-intruder test (attack frequency: two-way ANOVA, drug F(1, 17) = 0.3570, P = 0.5581; genotype F(1, 17) = 17.12, P = 0.0007; interaction F(1, 17) = 0.9182, P = 0.3514). Data are presented as mean ± SEM. *** represents P<0.001.

Cycling of mania and depression is a characteristic feature of bipolar disorder for which the drug of choice is lithium [40]–[42]. Since, hyperaggression- and depression-like behavior in the GluD1 KO mirror some aspects of bipolar disorder, we tested the effect of chronic lithium on these behaviors [43]. Four groups of mice, control diet WT, lithium diet WT, control diet GluD1 KO and lithium diet GluD1 KO were kept on a lithium treatment protocol as described in the *Methods*. We first performed the open field test (n = 7–9 for each group). We found no drug X genotype effect in the open field test (Fig. 5A; two-way ANOVA, drug F(1, 27) = 0.8275, P = 0.3710; genotype F(1, 27) = 5.678, P = 0.0245; interaction F(1, 27) = 2.589, P = 0.1192). We next performed the forced swim test (n = 11–13 for each group). Lithium was effective in normalizing the depression-like behavior as measured by duration of immobility in the forced swim test (Fig. 5B; two-way ANOVA, drug F(1, 43) = 9.717, P = 0.0033; genotype F(1, 43) = 80.49, P<0.0001; interaction F(1, 43) = 15.84, P = 0.0003). Thus lithium was able to rescue depression-like behavior in GluD1 KO. Further we performed the resident-intruder test using a different set of animals (n = 5–6 for each group). Lithium was not effective in reversing the hyperaggression as measured by number of attacks (Fig. 5C; two-way ANOVA, drug F(1, 17) = 0.3570, P = 0.5581; genotype F(1, 17) = 17.12, P = 0.0007; interaction F(1, 17) = 0.9182, P = 0.3514).

### GluD1 knockout mice exhibit behavioral abnormalities in social interaction and D-Cycloserine rescues the social interaction deficit in GluD1 knockout mice

**Figure 6:**
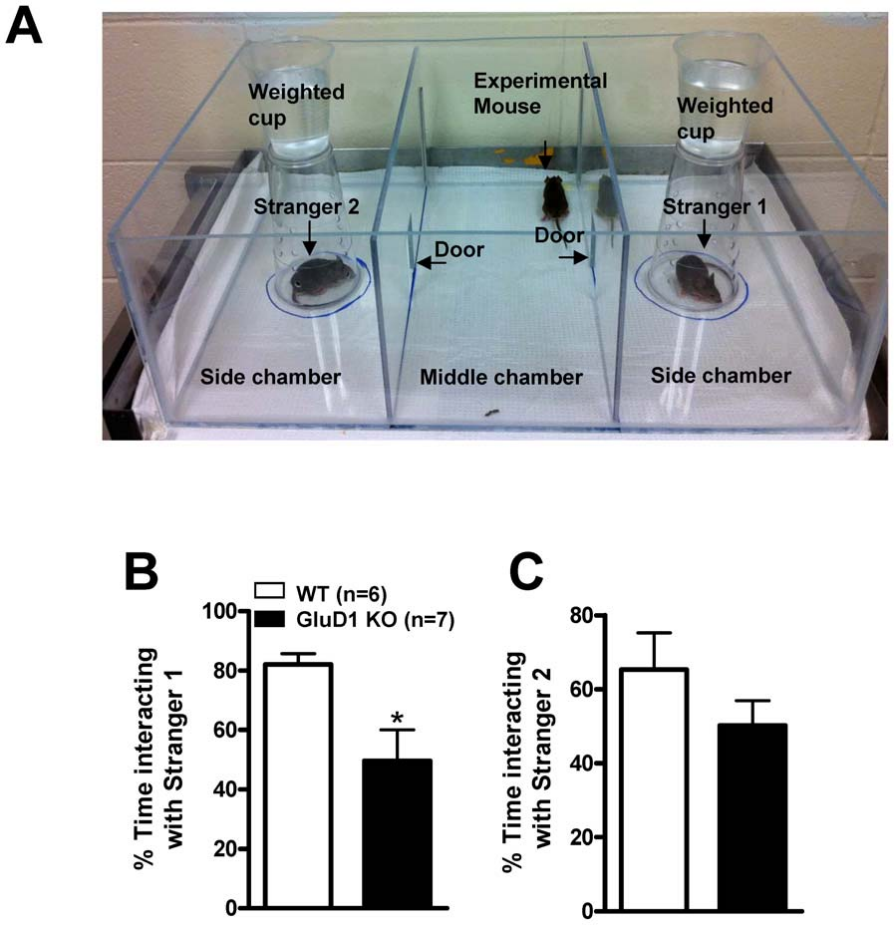
GluD1 KO exhibit a deficit in social interaction. **A.** The social interaction chamber with the experimental and stranger mice. **B.** In the sociability test GluD1 KO mice (n = 7) spent less percent time interacting with the Stranger 1 (unpaired t-test, P = 0.0215, F = 9.851) compared to WT mice (n = 6). **C.** In the test for social novelty where a second stranger (Stranger 2) mouse was introduced into the empty container there was no significant difference in the percent time spent interacting with Stranger 2 in WT and GluD1 KO mice (unpaired t-test, P = 0.2420, F = 1.904). Data are presented as mean ± SEM. * represents P<0.05.

GRID1 gene is strongly associated with schizophrenia and ASD, disorders that are characterized by social deficits [44]–[47]. Therefore we next tested whether GluD1 KO exhibit social interaction deficits. We performed the sociability test as previously described [37], [38]. Figure 6A depicts a picture of the social interaction chamber with the experimental and stranger mice. In the test of social approach, where the mice chooses to explore either an empty container or a container with a conspecific mouse (Stranger 1), the WT mice (n = 6) spent more time interacting with Stranger 1 compared to the empty container (Fig. 6B). This is in accordance with a preference of a WT mouse for social interaction [48]. However, GluD1 KO mice (n = 7) spent almost the same time around the container with the Stranger 1 or the empty container suggesting that GluD1 KO have a deficit in social interaction (Fig. 6B). There was a significant difference in the percent time interacting with Stranger 1 in WT and GluD1 KO (Fig. 6B; unpaired t-test, P = 0.0215, F = 9.851). In the test for social novelty where a second mouse (Stranger 2) was introduced into the empty container there was no significant difference in the percent time spent interacting with Stranger 2 in WT and GluD1 KO mice (Fig. 6C; unpaired t-test, P = 0.2420, F = 1.904).

**Figure 7:**
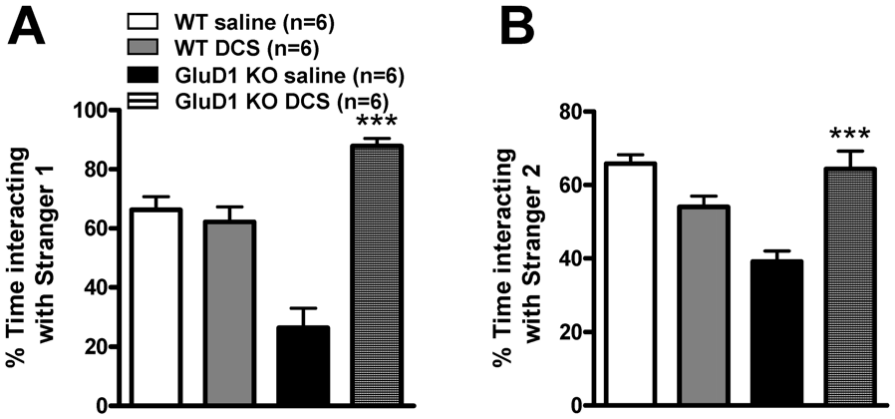
DCS rescues social interaction deficits in GluD1 KO. **A.** WT and GluD1 KO mice were intraperitoneally injected with DCS (320 mg/kg) or saline 20 min prior to sociability test (n = 6 for each group). DCS administration in GluD1 KO significantly enhanced percent time spent interacting with Stranger 1 compared to the empty container (two-way ANOVA, drug F(1, 20) = 34.28, P<0.0001; genotype F(1, 20) = 2.103, P = 0.1625; interaction F(1, 20) = 44.79, P<0.0001). **B.** In the test of social novelty GluD1 KO showed significantly higher percent time interacting with Stranger 2 compared to Stranger 1 (two-way ANOVA, drug F(1, 20) = 3.897, P = 0.0624; genotype F(1, 20) = 5.811, P = 0.0257; interaction F(1, 20) = 29.66, P<0.0001). Data are presented as mean ± SEM. *** represents P<0.001.

DCS is an NMDA receptor GluN1 subunit agonist [49] that has shown efficacy in social interaction behaviors in rodents and social impairment in humans [28], [50], [51]. We therefore tested the effect of DCS on the social deficits in GluD1 KO mice. WT and GluD1 KO mice were intraperitoneally injected with DCS (320 mg/kg) or saline 20 min prior to sociability test (n = 6 for each group). Remarkably, DCS administration in GluD1 KO significantly enhanced percent time spent interacting with Stranger 1 compared to the empty container (Fig. 7A; two-way ANOVA, drug F(1, 20) = 34.28, P<0.0001; genotype F(1, 20) = 2.103, P = 0.1625; interaction F(1, 20) = 44.79, P<0.0001). In the test of social novelty, DCS administered GluD1 KO showed significantly higher percent time interacting with Stranger 2 compared to Stranger 1 (Fig. 7B; two-way ANOVA, drug F(1, 20) = 3.897, P = 0.0624; genotype F(1, 20) = 5.811, P = 0.0257; interaction F(1, 20) = 29.66, P<0.0001). Additionally, a small group of GluD1 KO mice, that received a single dose of DCS (320 mg/kg) were retested after 2 weeks of the first test and demonstrated normal social interaction (data not shown). This data suggests that a single dose of DCS may have long lasting effects. Of note, the differences in the values for the saline treated mice (Fig. 7A, B) versus naïve mice (Fig. 6B) in the social interaction test may arise due to the injection stress.

### Molecular abnormalities in the amygdala and prefrontal cortex of GluD1 knockout mice and effect of D-Cycloserine

**Figure 8:**
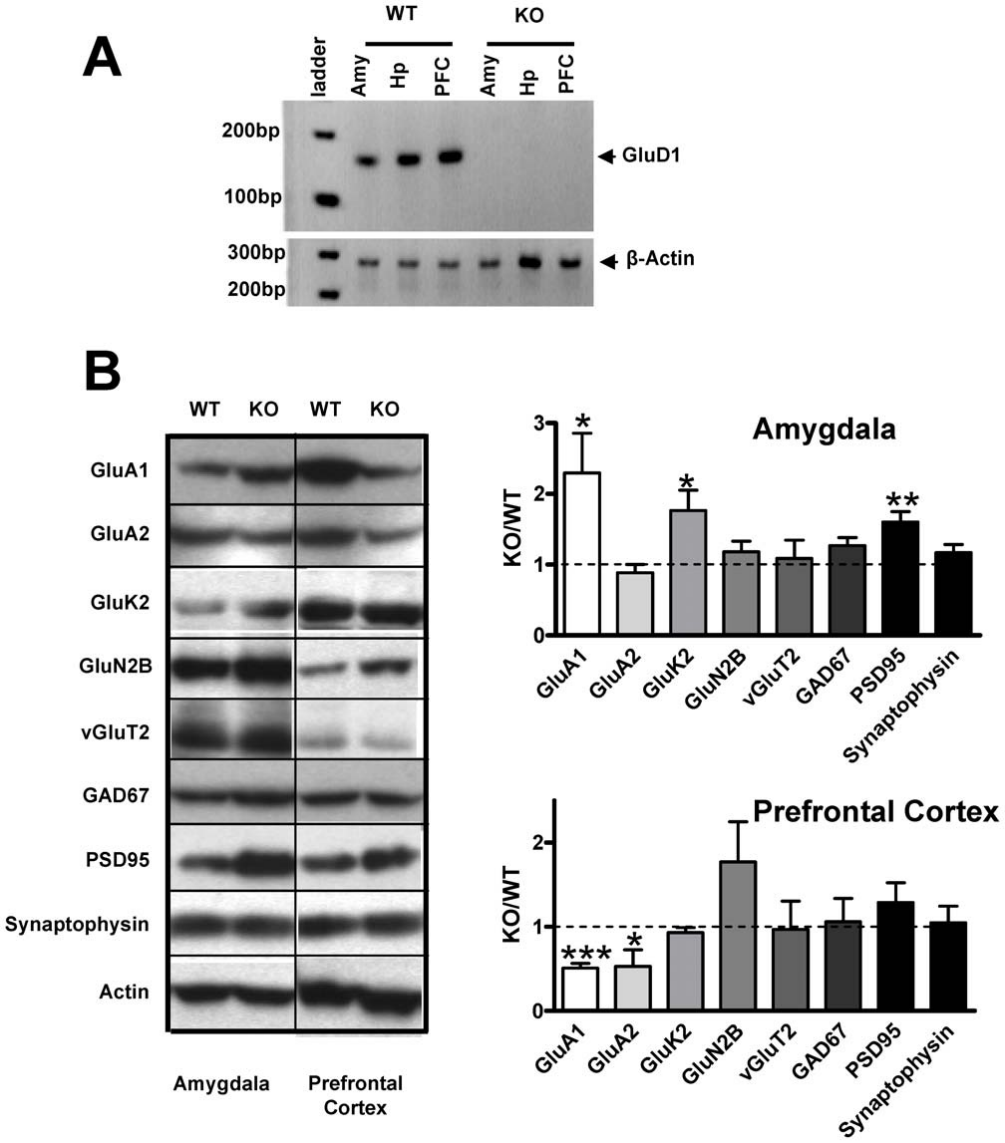
Altered expression of iGluR subunits and synaptic proteins in GluD1 KO. **A.** RT-PCR showed expression of the GluD1 mRNA in the amygdala, hippocampus and the prefrontal cortex of P15 WT mice. No expression of GluD1 mRNA was seen in GluD1 KO. **B.** Synaptoneurosomes were collected (5–11 animals each for WT and GluD1 KO) and western blotting analysis was performed for iGluR subunits and synaptic proteins. In synaptoneurosomal preparations from the amygdala of GluD1 KO and WT mice we found a significantly higher expression of GluA1 (P = 0.0486), GluK2 (P = 0.0327) and PSD95 (P = 0.0028) and a trend for higher expression of GAD67 (P = 0.0640) in GluD1 KO. In the prefrontal cortex we observed a significantly lower expression of GluA1 (P<0.001) and GluA2 (P = 0.0345) in GluD1 KO. Data are presented as mean ± SEM. *** represents P<0.001, ** represents P<0.01 and * represents P<0.05.

Next we determined if expression of other iGluR subunits and synaptic proteins is altered in the GluD1 KO mice. We specifically tested changes in amygdala and prefrontal cortex of GluD1 KO since these regions have been shown to regulate social and emotional behaviors [52]–[55] . As a first step we performed RT-PCR to determine expression of GluD1 signal in amygdala and prefrontal cortex. GluD1 mRNA could be detected in both amygdala and prefrontal cortex by RT-PCR, in addition to hippocampus which is the primary site for GluD1 expression [1], [10] (Fig. 8A). Synaptoneurosomes were collected for 5–11 animals each for WT and GluD1 KO. We tested the effect of deletion of GluD1 on a set of proteins representative of; (1) iGluR subunits: GluA1, GluA2, GluK2, GluN2B; (2) presynaptic and postsynaptic proteins: synaptophysin and PSD95 respectively and (3) excitatory and inhibitory neurons: vGluT2 and GAD67 respectively. In the amygdala we found a significantly higher expression of GluA1 (P = 0.0486), GluK2 (P = 0.0327) and PSD95 (P = 0.0028) and a trend for higher expression of GAD67 (P = 0.0640) in GluD1 KO (Fig. 8B, Table 1). A significantly lower expression of GluA1 (P<0.001) and GluA2 (P = 0.0345) was observed in the prefrontal cortex of GluD1 KO (Fig. 8B, Table 1). These results suggest that deletion of GluD1 leads to significant changes in the expression of synaptic proteins which are crucial for both excitatory and inhibitory synaptic neurotransmission.

**Table 1:** Synaptic protein composition in GluD1 KO mice.

Protein	Amygdala			Prefrontal Cortex		
	Ratio	SEM	P	Ratio	SEM	P
**GluA1**	2.293	0.563	0.0486*	0.508	0.056	<0.001***
**GluA2**	0.885	0.115	0.5686	0.528	0.198	0.0345*
**GluN2B**	1.180	0.152	0.2780	1.770	0.478	0.1364
**GluK2**	1.764	0.286	0.0327*	0.930	0.060	0.4428
**vGluT2**	1.087	0.258	0.7556	1.059	0.277	0.8747
**GAD67**	1.269	0.111	0.0640	0.941	0.207	0.8027
**PSD95**	1.601	0.146	0.0028**	1.286	0.237	0.2761
**Synaptophysin**	1.166	0.118	0.2905	1.049	0.196	0.8176

Synaptoneurosomes were isolated from the GluD1 KO and WT amygdala and prefrontal cortex and western blotting was performed (5–11 animals for each group). Data are presented as mean ± SEM. First, the optical density of each sample was normalized to β-actin. Thereafter, the optical density was normalized to the mean of the WT samples. The average ± SEM of optical densities of GluD1 KO samples, that were normalized to WT mean, are represented as Ratio (KO/WT) ± SEM. The P values were calculated from optical densities of WT and GluD1 KO samples normalized to the WT mean.

*** represents P<0.001.

** represents P<0.01 and.

* represents P<0.05.

**Figure 9:**
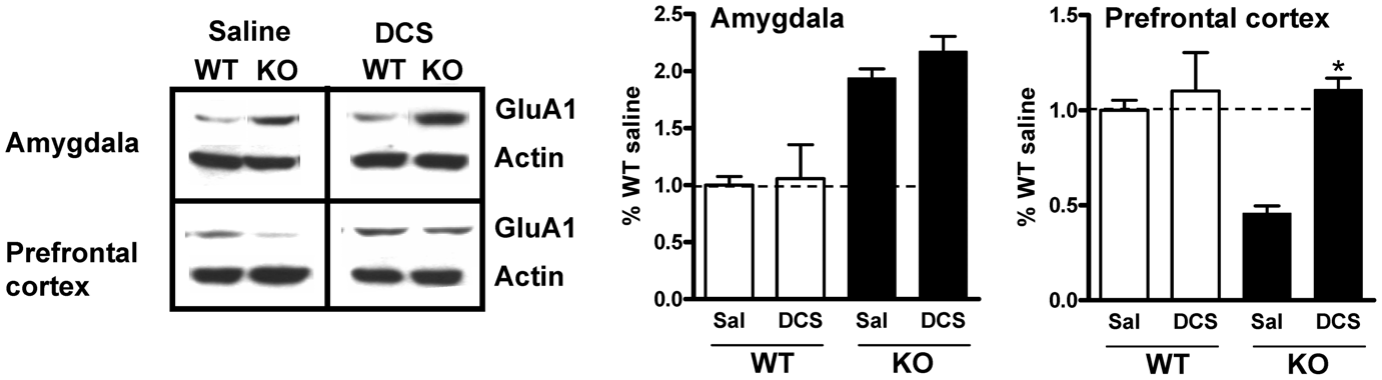
DCS normalized the reduced GluA1 expression in prefrontal cortex in GluD1 KO. Synaptoneurosomes were prepared from amygdala and prefrontal cortex 2 hours after the end of the social novelty test in which saline or DCS (320 mg/kg) was administered intraperitoneally (Fig. 7) (n = 3 for each group). GluA1 expression was higher in GluD1 KO amygdala and lower in the prefrontal cortex. DCS did not normalize the higher GluA1 expression in amygdala (two-way ANOVA, drug F(1,8) = 0.7175, P = 0.4216; genotype F(1,8) = 35.59, P = 0.0003; interaction F(1,8) = 0.2622, P = 0.6224). In the prefrontal cortex the lower GluA1 expression was increased to the WT levels by DCS treatment (two-way ANOVA, drug F(1,8) = 11.57, P = 0.0093; genotype F(1,8) = 5.884, P = 0.0415; interaction F(1,8) = 6.140, P = 0.0382). Data are presented as mean ± SEM. * represents P<0.05.

We further tested whether the effect of DCS on social interaction deficit is mediated by normalization of altered synaptic proteins. We focused on GluA1 expression since this was altered in both amygdala and prefrontal cortex. Synaptoneurosomes were prepared from amygdala and prefrontal cortex, 2 hours after the end of social novelty test in which saline or DCS (320 mg/kg) was administered (n = 3 for each group). As seen in Fig. 9, DCS did not normalize the higher GluA1 expression in amygdala (Fig. 9, two-way ANOVA, drug F(1,8) = 0.7175, P = 0.4216; genotype F(1,8) = 35.59, P = 0.0003; interaction F(1,8) = 0.2622, P = 0.6224). However, in the prefrontal cortex the lower GluA1 expression was increased to the WT levels by DCS treatment (Fig. 9, two-way ANOVA, drug F(1,8) = 11.57, P = 0.0093; genotype F(1,8) = 5.884, P = 0.0415; interaction F(1,8) = 6.140, P = 0.0382). These results are in accordance with a potential role of glutamate receptors in the prefrontal cortex in social behaviors [53], [56], [57].

## Discussion

Among the ionotropic glutamate receptor family, GluD1 is the least studied and its role in the CNS remains unknown. We have identified a novel role of GluD1 whereby its deletion leads to appearance of aberrant behaviors that mirror emotional and social behaviors in patients with neuropsychiatric disorders. In particular, we observed that deletion of GluD1 led to hyperactivity, lower anxiety-like behavior, depression- and aggression-like behavior and social interaction deficits. These deficits were partially rescued by treatment with lithium and D-Cycloserine (DCS). Since amygdala and prefrontal cortex have been implicated in these emotional and social behaviors [52], [58]–[61], we hypothesized that GluD1 deletion may lead to abnormalities in these regions. Indeed, we observed changes in the expression of synaptoneurosomal proteins in both amygdala and prefrontal cortex.

### Behavioral abnormalities due to deletion of GluD1

One of the unique features of GluD1 knockout was the presence of both depression- and aggression-like behaviors (Fig. 3, 4) which mirror some features of bipolar disorder-like behavior. We therefore tested whether lithium, a mood stabilizer, may be effective in these behaviors. Lithium was effective in reducing immobility in the forced swim test in GluD1 knockout (Fig. 5B). However, lithium was ineffective in hyperactivity (Fig. 5A) and aggression-like behavior in the resident-intruder test (Fig. 5C). We also observed significant deficits in the sociability test in the GluD1 knockout which were rescued by DCS treatment (Fig. 6B, 7A, B). DCS has been shown to improve impaired sociability in Balb/c mice [28] and enhance pair bonding in prairie voles [50]. In humans, DCS has also been found to improve social withdrawal in autistic patients [51] and enhance exposure therapy in social anxiety disorder [62], [63]. Our study is in agreement with this growing body of evidence that DCS may be effective in treating social interaction deficits. At the molecular level we found that DCS reversed the lower GluA1 expression in the prefrontal cortex but did not normalize the higher GluA1 expression in amygdala (Fig. 9). These data suggest that the effect of DCS on social interaction behavior may be mediated by affecting signaling in the prefrontal cortex. DCS is an agonist at the NMDA receptor and its efficacy, in comparison to endogenous agonists' glycine and D-serine is GluN2 subtype dependent. Specifically, DCS is a partial agonist with ∼60% efficacy at GluN2B receptors but acts as a full agonist at other GluN2 subtypes [49], [64]. Thus, one possibility is that the effect of DCS in GluD1 knockout on social interaction and GluA1 expression in the prefrontal cortex may arise due to the inhibition of GluN2B receptors. This hypothesis is in agreement with a recent study demonstrating that administration of a specific GluN2B inhibitor elevates GluA1 expression and leads to synaptogenesis in the prefrontal cortex [65]. It will therefore be of interest to test whether deletion of GluD1 leads to abnormal synaptogenesis in the prefrontal cortex and whether this can be rescued by DCS or GluN2B inhibitors.

We also found that GluD1 heterozygous mice when tested in the forced swim test had significantly higher immobility time (data not shown). These results further suggest that GluD1 subunit plays a non-redundant function in the CNS and the presence of both copies of GluD1 is necessary for normal behavior. Thus, lower expression of GluD1 may act as a predisposing factor for neuropsychiatric disorders. Interestingly, lower GluD1 expression has been reported in the cortex of human patients with schizophrenia, bipolar disorder and ASD [18], [66]. Additionally, in a chronic mild stress model of depression-like behavior in rats, GluD1 mRNA in the frontal cortex is downregulated and this could be completely reversed by the antipsychotic quetiapine [20], [21].

### Abnormalities in the expression of synaptoneurosomal proteins by GluD1 deletion

The potential mechanism for changes in expression of iGluR subunits in GluD1 knockout may be gleaned from studies on GluD2 which is the closest homolog of GluD1. Specifically, we observed abnormalities in the expression of GluA1, GluA2 and GluK2 which have all been shown to either directly interact with GluD2 forming a heteromer or are regulated during surface trafficking by GluD2 [67]–[69]. Thus it is conceivable that GluD1 may similarly be crucial in regulation of these iGluR subunits in the amygdala and prefrontal cortex. We also observed higher expression of PSD95 in the amygdala in GluD1 knockout which correlates with elevated PSD95 reported in lateral amygdala in patients suffering from depression [70]. Both the amygdala and prefrontal cortex have been implicated in major depression [71]–[77]. Additionally, changes in synaptic proteins and glutamate neurotransmission in the amygdala and prefrontal cortex are associated with depression [70], [78]–[81]. We also observed a trend for higher GAD67, a marker for inhibitory neurons, in the amygdala. This suggests a potential dysregulation of both excitatory and inhibitory synapses in the GluD1 knockout. Interestingly, excitatory-inhibitory imbalance in prefrontal cortex and amygdala have been reported in schizophrenia and ASD animal models [82]–[84], disorders with which GluD1 has been associated.

Another unique feature of the protein expression study was the opposing effects in the expression of α-amino-3-hydroxy-5-methyl-4-isoxazolepropionic acid receptor (AMPA) and kainate subunits in amygdala versus prefrontal cortex. Broadly, there was an upregulation of expression in the amygdala and a downregulation in prefrontal cortex. Moreover, DCS selectively normalized GluA1 expression in the prefrontal cortex and lithium rescued depression-like behavior but not hyperactivity and aggression like behavior. These results suggest that deletion of GluD1 may have unique and contrasting effects on synapses in different brain regions which likely lead to these unique arrays of behavioral anomalies. Future electrophysiological and structural studies will be required to address the ability of GluD1 to selectively modify neural circuitry in different brain regions.

One of the shortcomings in our molecular studies is that we did not analyze other regions in the brain that may be involved in emotional or social behaviors, a prominent one being the hippocampus. Decreased hippocampal volume and moderate apoptosis has been reported in the hippocampus in patients with major depression [67]–[74]. Additionally, aggressive behavior results in reduction in hippocampal volume [75], [76]. The hippocampus also has a role in social behaviors. Hippocampal lesions in rodents lead to abnormal social behavior and social interaction [77]–[79]. Studies in humans have also reported differential activity in neurons within the hippocampal formation during presentation of social cues [80]–[82]. The hippocampus also has a role in anxiety-like behavior [83], [84]. On a molecular level post-mortem studies have reported a decrease in AMPA and kainate receptor expression in the hippocampus in schizophrenia, bipolar disorder and major depression [85]–[91]. Studies using mouse models have demonstrated a role of AMPA receptors in the hippocampus in mania- and depression-like behavior [92], [93]. In addition, GluK2 knockout mice exhibit mania-like behavior along with lower GluK1 receptor expression in the hippocampus [27]. Thus, abnormalities in hippocampal function and changes in AMPA/kainate expression in the hippocampus may be involved in the pathophysiology of certain types of mental disorders and behavioral abnormalities similar to those observed in GluD1 knockout. Given that GluD1 is highly expressed in the hippocampus it is possible that deletion of GluD1 may lead to synaptic abnormalities including changes in AMPA/kainate receptor expression in the hippocampus. Further studies are therefore necessary to delineate the role of hippocampus in the behavioral abnormalities observed in GluD1 knockout. Additionally, since the pharmacology of GluD1 is poorly understood [85], the best tool to study the brain region-specific role of GluD1 would be through selective genetic manipulations.

### GluD1 dysfunction and relevance to synaptic theory of mental disorders

As previously stated, the GRID1 gene that codes for GluD1 is strongly associated with mental disorders including schizophrenia, bipolar disorder, ASD and major depression [14]–[26], [94]–[96]. Our behavioral studies in GluD1 knockout further strengthens the idea that dysfunction of GluD1 may lead to behavioral deficits. A growing body of evidence suggests that disorders like schizophrenia, bipolar disorder and ASD which show genetic overlap [97] involve synaptic abnormalities that predispose individuals to these conditions. Mutations affecting a number of synaptic proteins have been observed in schizophrenia, bipolar disorder and ASD [98]–[103]. GluD1 is developmentally expressed and has been shown to affect presynaptic differentiation and synapse formation in *in vitro* systems [8]. Additionally, abundant data exists on the role of GluD2, which is the closest homolog of GluD1, in synapse formation [5], [6], [8], [67]. Moreover, it was recently shown that postsynaptic GluD2 interacts with presynaptic neurexin1 via cerebellin 1 protein [13] and a similar interaction may occur with GluD1. This finding is particularly interesting since mutations in neurexins are well known to be associated particularly with schizophrenia and ASD [22], [104]–[106]. These converging human genetic and molecular studies on GluD1 together with our results suggest that GluD1 might be a synaptic protein whose dysfunction may lead to abnormal synapse formation or function which predisposes individuals to neuropsychiatric conditions. Indeed further experiments are required to identify a specific role of GluD1 in the regulation of synaptic structure or function. Moreover, these novel roles may provide valuable insights into the regulation of behaviors relevant to neuropsychiatric disorders.

Animal models for mood disorders and other mental disorders are important for exploring the pathophysiology of human disease and for development of treatments. Animal models exhibiting spontaneously alternating mania- and depression-like behaviors for bipolar disorder are not known [107]. Among the prevailing models for bipolar disorder are overexpression of glycogen synthase kinase 3 beta (GSK3 beta) [108] and clock mutant [109]. Interestingly, the occurrence of both mania- and depression-like behaviors suggests that GluD1 knockout may have some face validity for bipolar-like behavior. Additionally lithium was able to rescue the depression-like behavior in GluD1 knockout suggesting that the model may have some predictive validity. Further studies will however be required to address whether there is any altering pattern of behaviors and to further establish predictive and construct validity. It is also possible that the behavior exhibited by GluD1 knockout may relate more closely to other mental disorders specifically ASDs and further detailed studies will be required to test this hypothesis.
